# Tamoxifen sensitivity-related microRNA-342 is a useful biomarker for breast cancer survival

**DOI:** 10.18632/oncotarget.21577

**Published:** 2017-10-06

**Authors:** Jessica Young, Tsutomu Kawaguchi, Li Yan, Qianya Qi, Song Liu, Kazuaki Takabe

**Affiliations:** ^1^ Breast Surgery, Department of Surgical Oncology, Roswell Park Cancer Institute, Buffalo, NY 14263, USA; ^2^ Department of Biostatistics & Bioinformatics, Roswell Park Cancer Institute, Buffalo, NY 14263, USA; ^3^ Department of Surgery, University at Buffalo Jacobs School of Medicine and Biomedical Sciences, The State University of New York, Buffalo, NY 14203, USA

**Keywords:** breast cancer, tamoxifen, response, prognostic biomarker, microRNA

## Abstract

MicroRNAs (miRNAs) have emerged as one of the crucial regulators of cancer progression. Some miRNAs are reported to be related to the response of breast cancer to tamoxifen (TAM). In this study, we investigated whether the levels of TAM response-related miRNAs translate to patient survival. The Cancer Genome Atlas (TCGA) and Gene Expression Omnibus (GEO) datasets were used and Gene Set Enrichment Analysis (GSEA) was performed. Four TAM response-related miRNAs, miR-221, miR-222, miR-342, and miR-451, were identified by literature search. Patients with high expression of miR-342, related to TAM sensitivity, were associated with better survival in TCGA cohort (Overall Survival (OS), p=0.02; Disease Free Survival (DFS), p=0.03, respectively), and in two other independent GEO cohorts (OS, p=0.02 and p=0.0007, respectively). High expression of miR-342 was associated with significantly better survival in ER-positive patients (p=0.04), but not in ER-negative or triple-negative patients. Surprisingly, high expression of miR-451, reported to increase the sensitivity to TAM, was associated with worse survival (p=0.002). MiR-221 and miR-222 did not show any significance in survival. Lastly, GSEA demonstrated that lower miR-342 expression was significantly associated with the enrichment of TAM resistance-related gene expression, and higher miR-342 expression with TAM sensitivity-related gene expression, but miR-221, miR-222 and miR-451 were not. For the first time, we used “big data” from TCGA and GEO cohorts to analyze multiple miRNAs with respect to survival impact and TAM sensitivities. We demonstrated that TAM sensitivity-related miR-342 could be a promising biomarker, especially in luminal type breast cancer patients.

## INTRODUCTION

Over 40,500 women in the US are still anticipated to die from breast cancer in 2017 [1, 2]. Most of these deaths will occur in estrogen receptor (ER)-positive breast cancers patients, despite its favorable profile, as it is the most common subtype [3, 4]. Selective estrogen receptor modulators (SERM), namely tamoxifen (TAM), and aromatase inhibitors are the mainstay of systemic therapy for this subtype. The Early Breast Cancer Trialists’ Collaborative Group showed that 5 years of adjuvant SERM treatment reduced the risk of disease recurrence by 41% and death by 34% in ER-positive early stage breast cancer patients [5]. But, their recurrence patterns are different from more aggressive subtypes, and it is not uncommon to see reemergence of disease after decades of dormancy. One of the mechanisms of this reemergence and mortality is TAM resistance [6]. To date, there is no widely accepted biomarker to identify resistance or sensitivity to TAM, which would allow us to avoid prescribing ineffective drugs or to counteract resistance. Measures to overcome TAM resistance are expected to be the most practical solution for improvement of the long term survival of ER-positive breast cancer patients.

Recently, microRNAs (miRNA), which are noncoding RNAs consisting of 22-25 nucleotides, have emerged as crucial new players in epigenetic regulation through the targeting of messenger RNA sequences in breast cancer [7–9]. Altered expression of miRNAs has been used for tumor diagnosis, staging, and prognostic biomarkers [10, 11]. In fact, it has been postulated that acquisition of drug resistance by cancer cells may be modulated through changes in miRNA levels. Therefore, identification of miRNAs that contribute to drug sensitivity or resistance is expected to provide us with new targets for therapeutic action.

Many tumor-related miRNAs have been reported as clinical biomarkers in breast cancer [12–16]. Alterations in miRNA levels contribute to cancer treatment response by either promoting mRNA degradation or suppressing its translation. However, most of the previous studies that described miRNAs as predictive biomarkers for drug resistance have been demonstrated in *in vitro* and/or *in vivo* systems or in a relatively small number of patient samples. Thus, in order to prove the utility of miRNAs as biomarkers, validation with larger clinical cohorts is needed.

To address this limitation, we utilized The Cancer Genome Atlas (TCGA), one of the largest collections of genomic cancer data, possessing both genetic and molecular information for over 1000 breast cancer cases with full clinical profiles and survival data [17, 18]. It is used to analyze cancers and understand how genomic changes drive disease forward, with the hope of enriching cancer prevention, detection and treatment. There are multiple reasons why TCGA cohort is an ideal database. First, it closely approximates national data for breast cancer distribution and survival [17, 18]. Second, the biospecimen samples need to meet a very stringent set of criteria to be used by advanced genomic analysis and sequencing technologies. Third, TCGA is a treatment-naïve cohort of over 1000 breast cancer patients, and therefore is a pure set of data to examine cells for biomarkers and their response to therapies. Fourth, it is also taken from fresh frozen samples, which is more accurate than paraffin embedded tissues [17, 18]. This is the same for some of the Gene Expression Omnibus (GEO) dataset tissue [19, 20]. Lastly, TCGA adopted the platforms of RNA-Sequence (RNA-Seq) and miRNA-Sequence (miRNA-Seq) for its determination of RNA expression, which has several advantages over the microarray platform. RNA-Seq does not need a “complementary probe” and it provides a practical base sequence for each RNA, whereas microarrays may show non-specific signals derived from false positive binding or probe reactions [21–23]. RNA-Seq and miRNA-Seq are expected to provide more precise analyses and identify genetic and epigenetic variations that were previously undetectable by microarray. Therefore, RNA-Seq has quickly become researchers’ preferred platform for transcriptome analysis [24].

The aim of this study is to use bioinformatics in a large clinical dataset to explore survival in patients with high expression of miRNAs reported to be related to TAM sensitivity or resistance. We conducted prognostic analyses using TCGA dataset, as well as other independent datasets retrieved from the GEO, to clarify whether TAM sensitivity- or resistance-related miRNAs show any survival impact in breast cancer patients. We also performed Gene Set Enrichment Analysis (GSEA) to validate whether each miRNA could be associated with known endocrine therapy response-related gene sets.

## RESULTS

### Literature search to identify candidate miRNA related to TAM sensitivity or resistance in breast cancer patients

A literature search was conducted using PubMed Central to identify miRNAs that were reported to relate to breast cancer TAM sensitivity or resistance. We identified several miRNAs of interest reported by multiple groups that lacked validation with a large patient cohort. Four miRNAs were selected for analysis; miR-221, miR-222, miR-342, and miR-451 [25–31] (Table 1). MiR-221 and miR-222 are reported to increase the resistance of breast cancer cells to TAM. MiR-342 and miR-451 are reported to increase the sensitivity of breast cancer cells to TAM. All previous reports utilized microarray or quantitative reverse transcription polymerase chain reaction (qRT-PCR) with cell lines for analysis, or had few patient samples.

**Table 1:** Candidates of endocrine therapy-related miRNAs based on literature search in breast cancer

miRNA	Target or related genes/pathways	Drug	Function	Materials	Platforms^1^	Reference
**miR-221/222**	CDKN1B	Tamoxifen	Resistance	Cell lines, primary tissue	Microarray, qRT-PCR	Miller, T (31)
	TGF-b signaling/Wnt signaling/ErbB signaling/Notch signaling/Jak-STAT signaling/MAPK signaling/p53 signaling/Focal adhesion	Fulvestrant	Resistance	Cell lines	Microarray, qRT-PCR	Rao, X (32)
	TIMP3	Tamoxifen	Resistance	Cell lines	qRT-PCR	Gan, R (33)
	ERa, P27	Tamoxifen	Resistance	Cell lines	qRT-PCR	Wei, Y (34)
**miR-342**	TXNIP, SEMAD, BMP7, GEMIN4	Tamoxifen	Sensitive	Cell lines, primary tissue	Microarray, qRT-PCR	Cittelly, DM (35)
	N/A	Tamoxifen	Sensitive	Cell lines	Microarray, qRT-PCR	Miller, T (31)
	Era	Tamoxifen	Sensitive	Cell lines	qRT-PCR	HE, YJ (36)
**miR-451**	YWHAZ	Tamoxifen	Sensitive	Cell lines	qRT-PCR	Bergamaschi, A (37)

qRT-PCR, quantitative reverse transcription polymerase chain reaction

### High expression of TAM sensitive miR-342 is associated with better survival in TCGA and GEO cohorts

**Figure 1:**
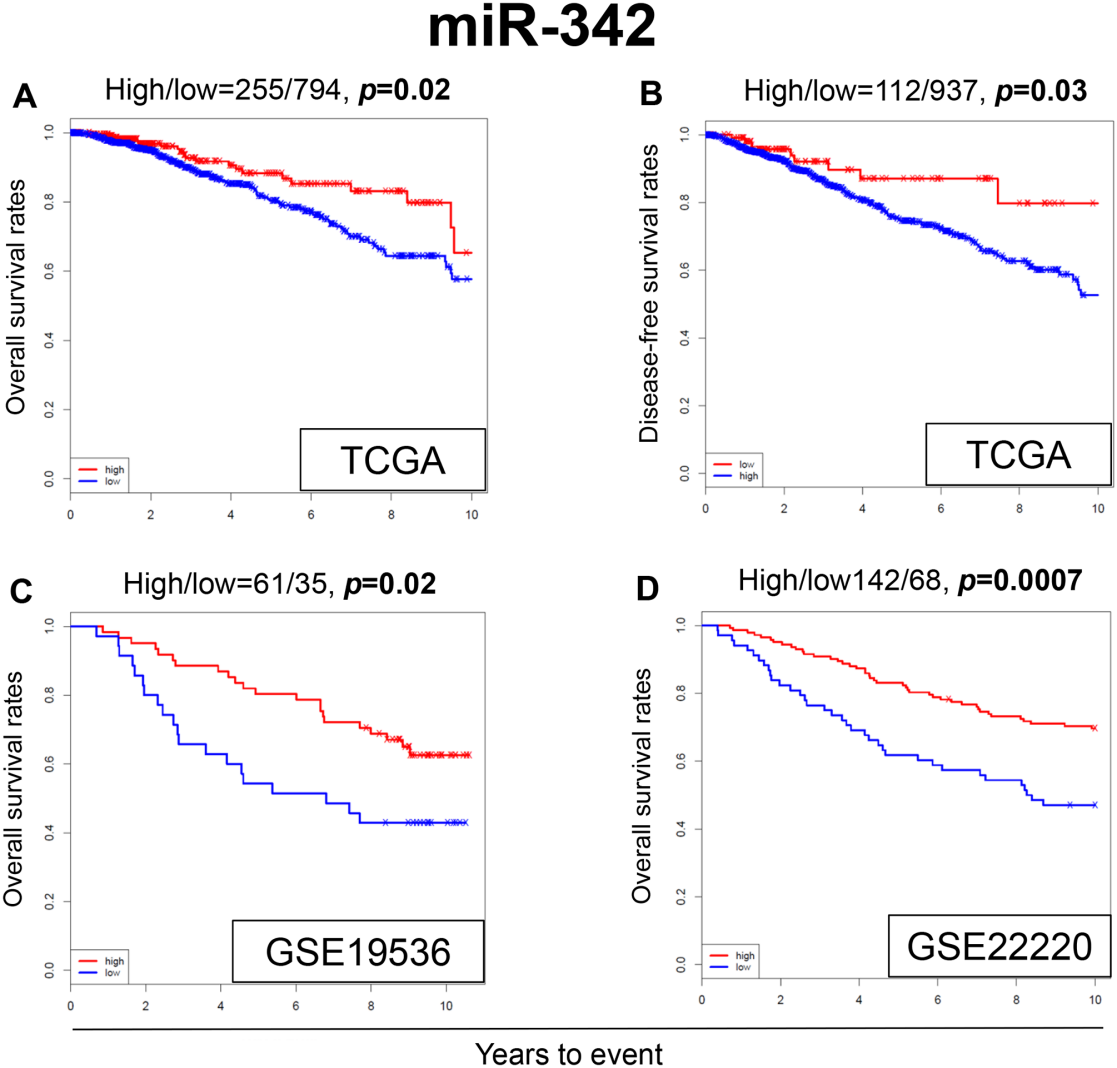
Expression levels of miR-342 and survival Kaplan-Meier estimates of survival among TCGA and GEO cohorts of breast cancer patients based on high and low expression of miR-342. **(A)** Overall survival (OS) in TCGA. **(B)** Disease-free survival (DFS) in TCGA. **(C)** OS in GSE19536. **(D)** OS in GSE22220. High and low expressions are represented by the red and blue lines, respectively. Bold font indicates significant difference. Not significant (N.S.).

High expression of miR-342 has been reported in the literature to be associated with TAM sensitivity in breast cancer *in vitro* [25, 29, 30]. Therefore, it was of interest to analyze its survival impact using a large clinical cohort. We found that patients with breast cancer that express high levels of miR-342 demonstrated significantly better overall survival (OS) as compared to patients with low expression (*p*=0.02) (Figure 1A). Patients with high expression of miR-342 were also noted to have significantly better disease free survival (DFS) compared to patients with low expression (*p*=0.03) (Figure 1B). Remarkably, similar results were observed in the two completely independent GEO cohorts, where breast cancer patients with high expression levels of miR-342 demonstrated significantly better OS in both cohorts (GSE19536, *p*=0.02; GSE22220, *p*=0.0007, respectively) (Figure 1C and 1D).

**Figure 2:**
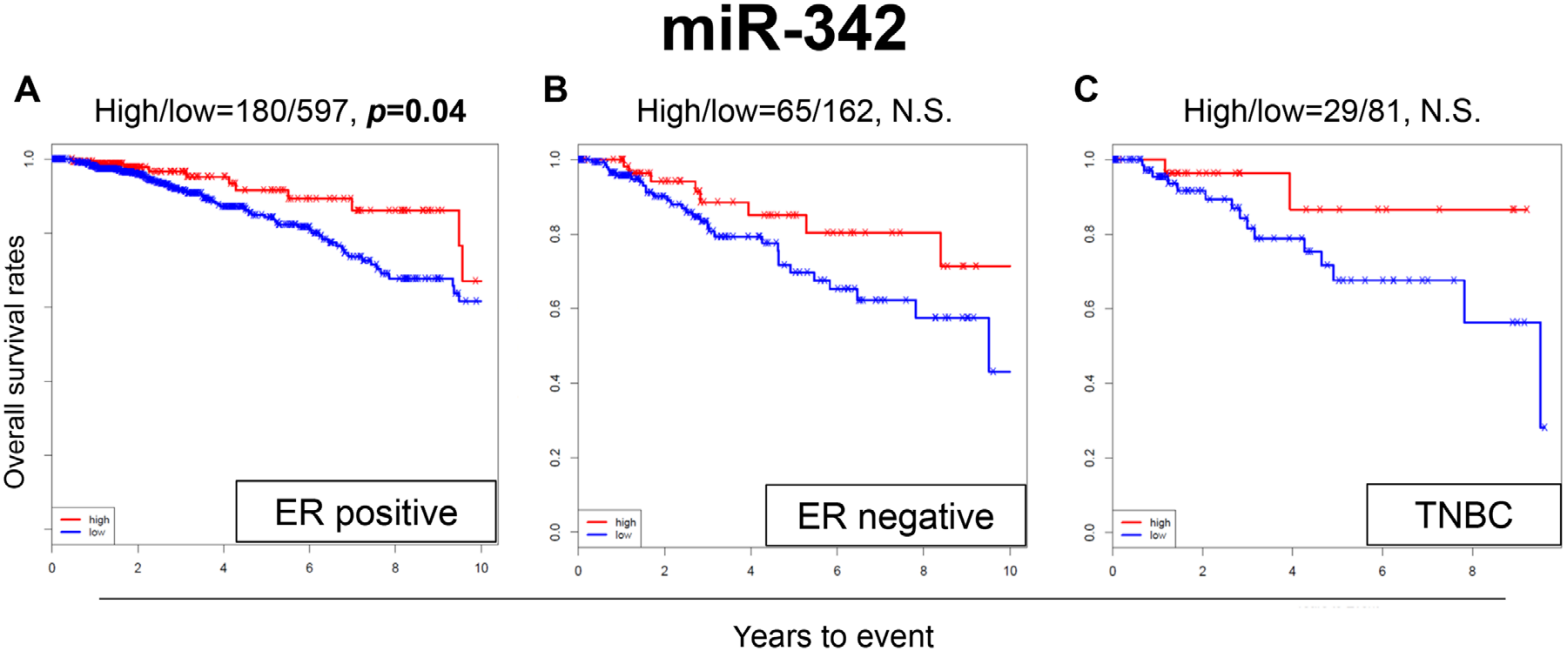
Expression levels of miR-342 and survival in **(A)** estrogen receptor (ER)-positive breast cancer population, **(B)** ER-negative breast cancer population, and **(C)** triple negative breast cancer (TNBC) population in TCGA. High and low expressions are represented by the red and blue lines, respectively. Bold font indicates significant difference. Not significant (N.S.).

When the OS was analyzed by different subtypes in TCGA cohort, patients with high expression levels of miR-342 demonstrated significantly better OS in ER-positive patients (*p*=0.04) (Figure 2A), whereas there was no significant difference in survival in ER-negative or triple-negative breast cancer (TNBC) patients (Figure 2B and 2C). We also conducted multivariable Cox proportional hazards regression analyses with the expression levels of miR-342 and other clinical or pathological factors, including age, stage, and ER/PR/HER2 status. The expression level of miR-342 was not identified as an independent prognostic factor in TCGA cohort (Supplementary Table 4).

Interestingly, several previous reports demonstrated that expression levels of miR-342 are associated with ERα expression levels in breast cancer tissue [30, 32–34]. We also found that miR-342 expression demonstrated the tendency to be associated with ER status in TCGA cohort (Table 2). Taken together, our results demonstrate that miR-342 contributes not only to ERα expression but also to TAM response in breast cancer patients that result in prolonged survival, and implicate it to be a useful prognostic biomarker in ER-positive breast cancer patients.

**Table 2:** Association between clinical or pathological factors and miR-342 expression levels

Variables	miR-342 ^1^		P-value ^2^
	High (n)	Low (n)	
Age <60	123	433	0.0927
>60	132	361	
Stage I/II/III/IV	45/140/66/4	133/468/177/16	0.5911
ER +/-	180/65	597/162	0.1100
PR +/-	150/95	525/233	0.0240
HER2 +/-	101/123	244/417	0.0370
TNBC Yes/No	29/195	81/579	0.8833

^1^ Cutoff point of this analyses is corresponding to optimal point derived from survival analyses.

^2^ Chi square test.

### High expression of miR-451 is associated with worse OS in TCGA cohort but not the GEO cohort

**Figure 3:**
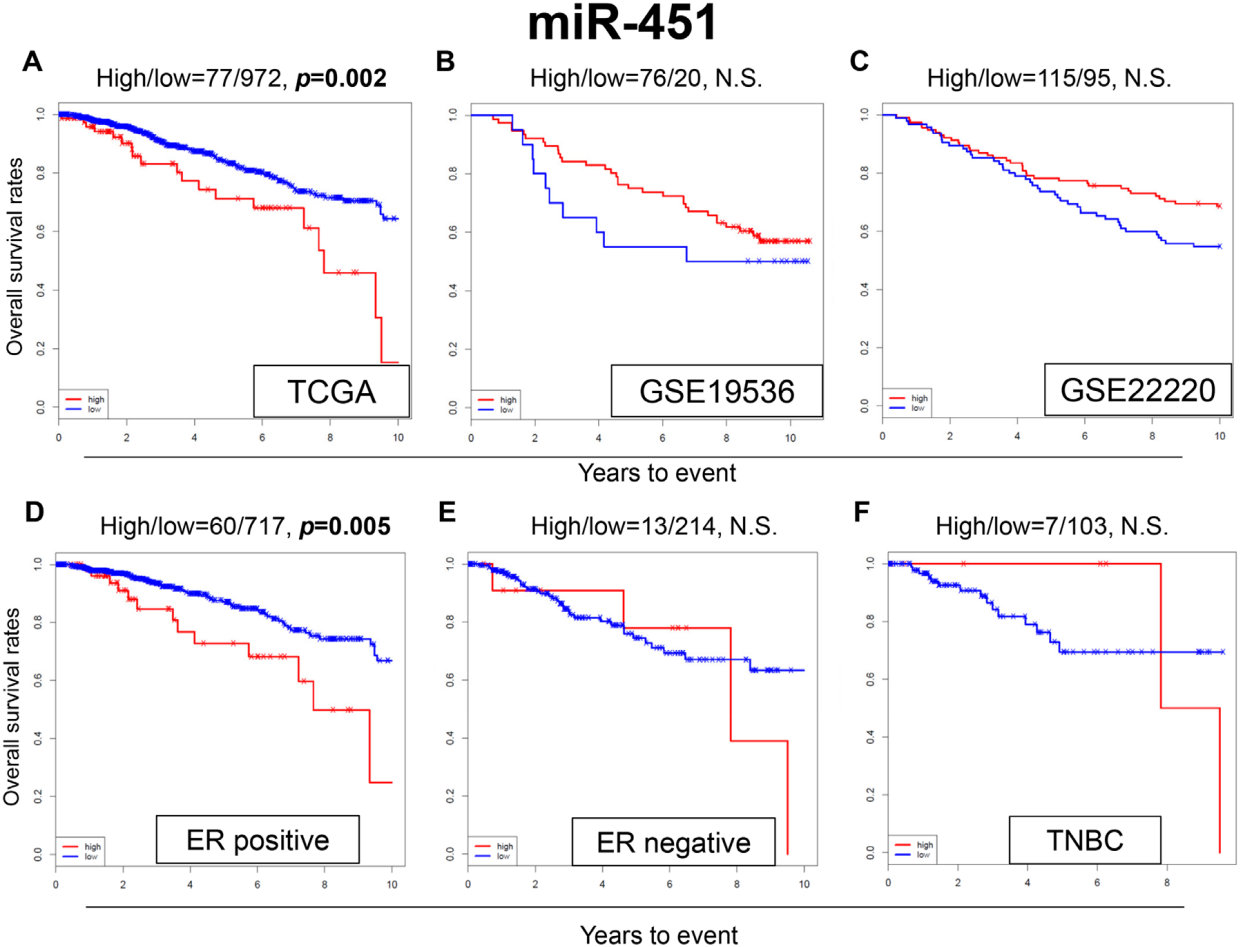
Expression levels of miR-451 and survival Kaplan-Meier estimates of survival among TCGA and GEO cohorts of breast cancer patients based on high and low expressions of miR-342. **(A)** OS in TCGA; **(B)** OS in GSE19536; **(C)** OS in GSE22220. Expression levels of miR-451 and survival in **(D)** ER-positive, **(E)** ER-negative and **(F)** TNBC breast cancer patients in TCGA. High and low expressions are represented by the red and blue lines, respectively. Bold font indicates significant difference. Not significant (N.S.).

MiR-451 was reported to increase the sensitivity to TAM in breast cancer cell lines [31]. Surprisingly, the patients with high expression of miR-451 were noted to have significantly worse OS compared to the patients with low expression in our analysis of TCGA data (*p*=0.002) (Figure 3A). However, we did not find any significant survival difference between high and low expression levels of miR-451 in the GEO cohorts (Figure 3B and 3C).

Among the ER-positive patients of TCGA cohort, patients with high expression of miR-451 were associated with worse OS (*p*=0.005) (Figure 3D), whereas no significant difference in OS was observed in the ER-negative and TNBC patients (Figure 3E and 3F). These results were the opposite of what we expected based on previous publications.

In addition, miR-451 expression levels did not show any significant association with clinical or pathological factors in TCGA cohort (Supplementary Table 1).

### MiR-221 and miR-222 expression levels are not associated with survival in TCGA and GEO cohorts

**Figure 4:**
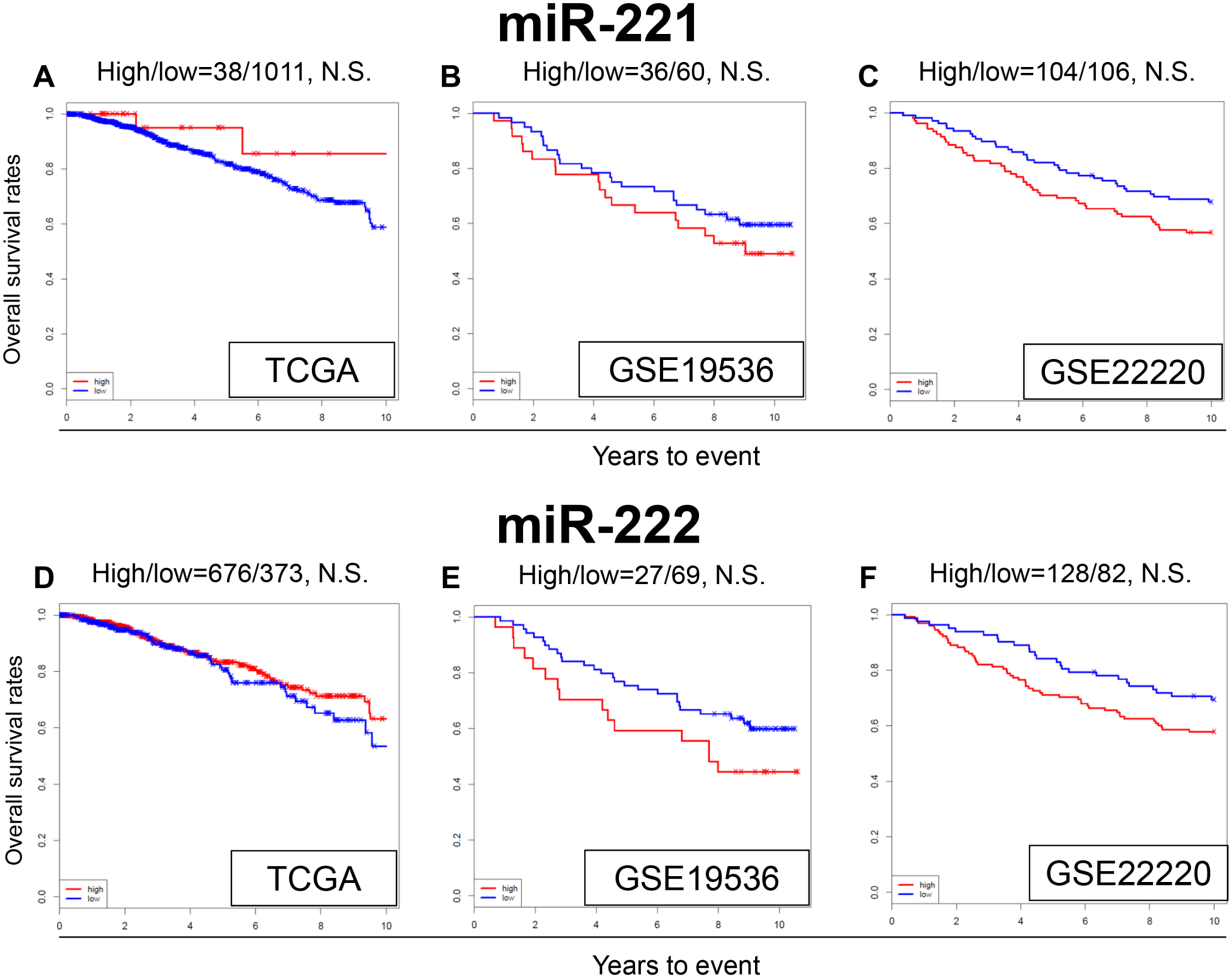
Expression levels of miR-221 and miR-222 and survival Kaplan-Meier estimates of survival among TCGA and GEO cohorts of breast cancer patients based on high and low expression of miR-221 and miR-222. OS for **(A)** miR-221, and **(D)** miR-222 in TCGA; OS for **(B)** miR-221 and **(E)** miR-222 in GSE19536; **(C)** OS for miR-221 **(F)** and miR-222 in GSE22220. High and low expressions are represented by the red and blue lines, respectively. Bold font indicates significant difference. Not significant (N.S.).

In the literature, miR-221 and miR-222 were reported to be associated with TAM resistance in breast cancer cells [25–28]. In our analysis, expression levels of miR-221 and miR-222 was not associated with OS in TCGA cohort (Figure 4A and 4D), or the GEO cohorts (Figure 4B-4E and 4F). MiR-221 and miR-222 did not show any significant difference in OS with high or low expression in any of the subtypes (Supplementary Figure 1). We were unable to reproduce the results previously seen in the literature, using these large patient cohorts.

In addition, miR-221 and miR-222 expression levels did not show any significant association with clinical or pathological factors in TCGA cohort (Supplementary Tables 2 and 3).

### The association between endocrine-therapy resistance or sensitivity and miRNA expression levels using GSEA

**Figure 5:**
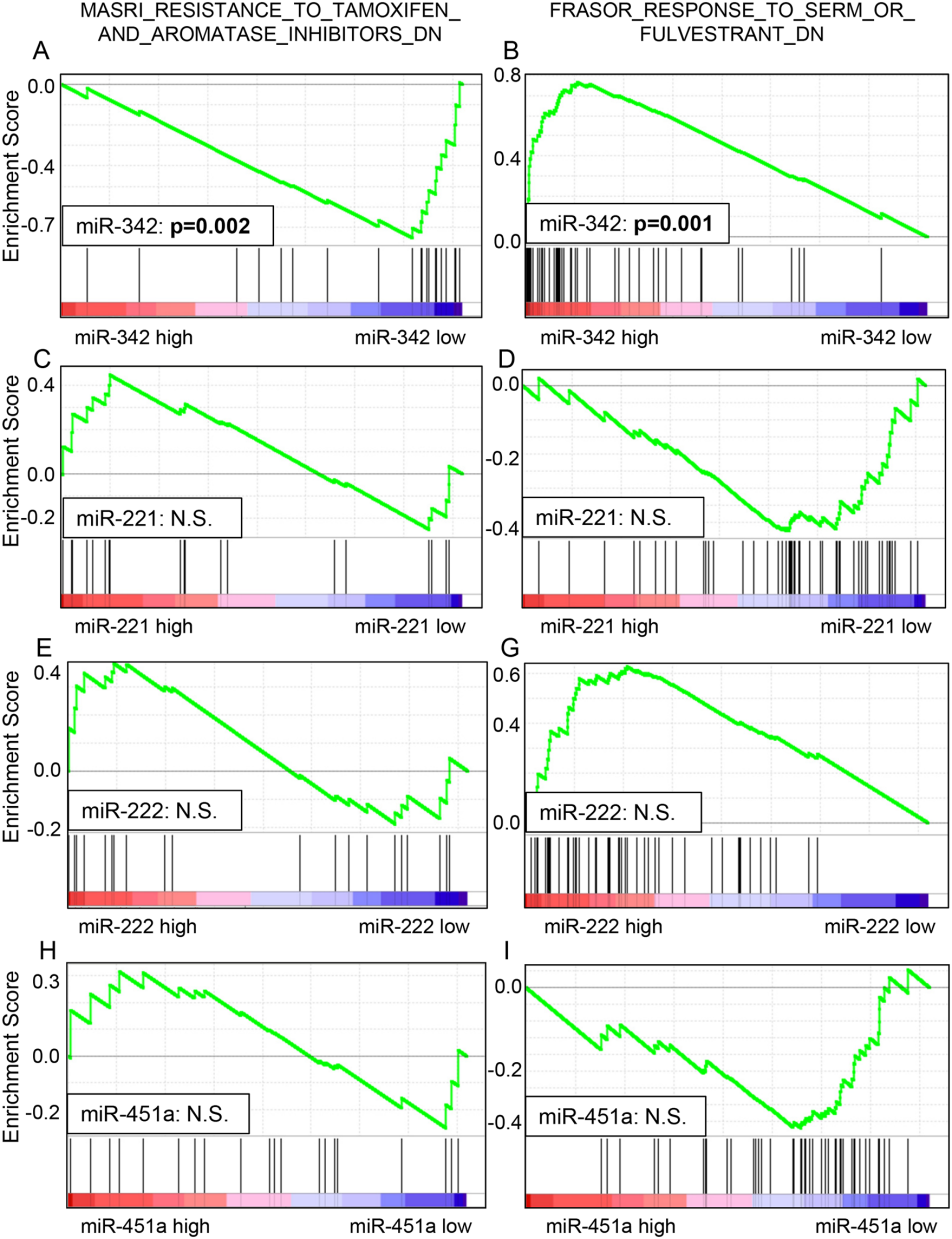
Gene set enrichment analysis (GSEA) of TCGA datasets showed that lower miR-342 expression was significantly associated with endocrine resistance in breast cancer **(A)**, and higher miR-342 expression was significantly associated with endocrine sensitivity in breast cancer **(B).** There was no significant enrichment noted in miR-221, miR-222, and miR-451 **(C-H)**.

GSEA of TCGA was performed to validate whether the miRNA expression levels correlated with TAM sensitivity or resistance. A predefined gene set from TCGA dataset that previously showed involvement in endocrine resistance (MASRI_RESISTANCE_TO_TAMOXIFEN_AND_AROMATASE_INHIBITORS_UP) was used [35]. We found that this gene set was significantly enriched in breast cancer with low expression levels of miR-342 (Enrichment Score (ES), -0.7514; Normalized ES, -1.6262; *p*=0.0020) (Figure 5A). Next, we used another predefined gene set from TCGA reported to be involved in SERM sensitivity (FRASOR_RESPONSE_TO_SERM_OR_FULVESTRANT_DN) [36]. We found that this gene set was significantly enriched in breast cancers with high expression levels of miR-342 (ES, 0.7629; Normalized ES, 1.9240; *p*=0.0018) (Figure 5B). These results demonstrate that genes related to TAM resistance were enriched with low expression of miR-342, and genes related to TAM sensitivity were enriched with high expression of miR-342. In contrast, we did not find any significant association between miR-221, miR-222 and miR-451 and the two gene sets listed above, with GSEA (Figure 5C-5H). The results are consistent with the survival analyses that showed expression of miR-221, miR-222 and miR-451 is not associated with sensitivity to TAM in breast cancer.

## DISCUSSION

The aim of our study was to examine whether expression of miRNAs reported to be related to TAM sensitivity or resistance is associated with patient survival in large clinical datasets. We expected that patients with high expression of miR-221 and miR-222, which was reported to be associated with TAM resistance, would have worse OS, whereas patients with high expression of miR-451 or miR-342, which was reported to be associated with TAM sensitivity, would have better OS. We used three large independent clinical datasets from TCGA and GEO to analyze survival with regards to these specific miRNAs. We found that high expression levels of miR-342 in breast cancer are associated with better OS, DFS, and OS in ER-positive subtypes. Through GSEA of two large TCGA gene sets, we also found that high expression of miR-342 enriched TAM sensitivity-related genes and low expression enriched TAM resistance-related genes. High expression of miR-451 unexpectedly demonstrated worse OS in our analysis of TCGA dataset, as well as decreased OS in the ER-positive subtype. We did not see the same trend in the GEO datasets, or through GSEA of TCGA. Expression levels of miR-221 and miR-222 did not show any significant association with survival in our analyses. To our knowledge, this is the first report to use TCGA or GEO to identify associations between miRNA expression levels and TAM sensitivity or resistance, and patient survival in large patient cohorts.

Higher levels of miR-342 expression have been reported to be associated with TAM sensitivity in the literature. Cittelly et al. found that downregulation of miR-342 is associated with TAM-resistant breast tumors, using cell lines and tissues from 16 breast cancer patients primary breast cancer [29]. Another group demonstrated that miR-342 expression was positively correlated with ERα mRNA expression in human breast cancer cell lines and proposed that it could predict TAM sensitivity in ERα-positive breast cancer and become a potential target for restoring ERα expression and response to antiestrogen therapy [30]. This result was validated by several groups [32–34]. In analyzing TCGA dataset for miR-342, we were able to corroborate these previous findings. High expression levels of miR-342 correlated to both increased OS in all three independent cohorts studied, as well as increased DFS in TCGA cohort. Therefore, high expression levels of miR-342 may be a superior prognostic biomarker in ER-positive breast cancer patients for TAM sensitivity and survival.

Bergamaschi et al. demonstrated that downregulation of miR-451 expression promoted breast cancer cell recurrence and endocrine resistance, thus concluding that high expression of miR-451 leads to TAM sensitivity [31]. This study was limited as it only used *in vitro* analyses of cell lines. The result of our survival analysis of TCGA cohort was opposite to our expectation based on this report. Miller et al. demonstrated that breast cancer cells overexpressing miR-221 or miR-222 had greater viability in the presence of TAM, and postulated that this conferred resistance to TAM through downregulation and loss of the effector molecule p27^Kip1^ [25]. Wei et al. also showed that secreted miR-221 or miR-222 could effectively reduce the target gene expression of p27 and ERα, which enhanced TAM resistance [28]. However, these reports are limited, having only *in vitro* data. Our study using large patient cohorts was unable to reproduce their results. Mechanisms for drug resistance remain difficult to elucidate, likely due to multiple concurrent mechanisms of action. Thus, it is difficult to explain why miR-221, miR-222 and miR-451 have shown some promise in pre-clinical studies, but not in our clinical dataset. With that said, given our data from large patient cohorts, it is safe to say that miR-221, miR-222 and miR-451 are not strong candidates for prognostic biomarkers at this time. On the other hand, miR-342 has shown promise as a biomarker both in preclinical studies from the literature, as well as in our analysis of 3 independent clinical datasets.

The limitations of our current study include only using retrospective data from TCGA and GEO datasets. Also, as there are no known absolute cutoffs and ranges available in this newer genomic data, these were generated after statistical analysis was done on the distributions of gene expressions within the datasets. Further, TCGA lacks many traditional clinicopathologic data points, which may have added to the analysis. However, despite these limitations, we feel that the associations found within our study are strong and valid, as we used a large clinical database for analysis.

In addition, we noticed that there was a large variation in the number of patients with high expression of miR-342.TCGA dataset had only 24.3% (255/1049) of breast cancer patients with high miR-342 expression out of 1,049 patients (24.3%), whereas 63.5% and 67.6% of patients showed high miR-342 expression in the GSE19536 and GSE2220 datasets, respectively. This difference may be attributed to multiple factors. First, the data originates from various populations in multiple countries, and therefore the outcomes data may be different. Also as described, the cutoff points between high and low expressions were determined by a running Cox proportional hazard statistic and not the number of patients or the absolute expression levels. Additionally, there was a lot of variation in the number of patients with high miR-451 seen among the datasets, as well as small numbers of patients with ER-negative or triple-negative tumors with high levels of miR-451. Despite the variation in numbers of patients in each group, we feel that by determining the threshold of dichotomization in the above manner [40], this statistical method is unbiased and statistically reliable [37] even though some small sample sizes were seen.

Recently there was a publication that stressed the importance of the translation of basic findings to clinical practice utilizing big data [37]. That aligns with the concept of this study to utilize bioinformatics to identify genomic and epigenomic prognostic biomarkers using publically available and established large cohorts such as TCGA and GEO. This concept is a good example of “Building Bridges between Basic and Clinical Genomic Research” in translational research [37].

In conclusion, for the first time, we used “big data” from TCGA and GEO cohorts to analyze multiple miRNAs with respect to overall survival and sensitivities to TAM. We found that high expression of miR-342 is strongly associated with overall survival and TAM sensitivity. Therefore, we conclude that miR-342 is a strong candidate as a biomarker to predict TAM sensitivity in breast cancer patients, and would have a clear clinical impact for treatment recommendations. On the other hand, the predicted outcomes for miR-221, miR-222, and miR-451 were not validated in our study. Future studies using large clinical datasets such as TCGA are expected to become increasingly important as we seek further clinical correlations with gene expression, leading to more biomarkers and actionable targets.

## MATERIALS AND METHODS

### Literature search to identify TAM sensitivity- or resistance-related miRNAs in breast cancer

We conducted a literature search using PubMed Central for articles from 2005 to 2016 that identified miRNAs that showed a relationship to TAM sensitivity or resistance in breast cancer. The criteria for selection were 1) the miRNA has demonstrated TAM sensitivity or resistance *in vitro* and/or *in vivo* in breast cancer; 2) the target mRNA or signaling pathway of the miRNA are identified in breast cancer; 3) the clinical relevance, including prognostic significance, of the miRNA has not been fully elucidated using large patient cohorts or multi-institutional clinical trials of breast cancer patients. Based upon this criteria, we analyzed the following miRNAs that have been reported to be related to tamoxifen sensitivity [12–16]; miR-10a, miR-30c, miR-31, miR-210, miR-326, miR-328, miR-487a, and miR-519a as well as miR-221, miR-222, miR-342 and miR-451. However, most of these miRNAs did not show any significant difference in survival. Therefore, only miR-342, miR-221, miR-222, and miR-451 were selected for further study.

### Extraction of miRNA expression levels and clinical dataset from TCGA and GEO

All data including miRNA-Sequence and clinicopathological data were retrieved from TCGA breast cancer cohort through the Genomic Data Common (GDC) portal, cBioportal, and Broad Institute Firehose. Survival data for the breast cancer patients in TCGA was obtained as previously reported [38]. In TCGA, 1049 samples were identified to have both miRNA-Seq and survival data, and the median observation period was 41.0 months (range, 0-286.8 months). We also found two independent breast cancer patient cohorts in the GEO that had miRNA expression data and survival information: GSE19536 (n = 96) and GSE22220 (n = 210) [19, 20]. Since TCGA and GEO are collections of de-identified, publically-accessible databases, Institutional Review Board Review was waived.

### Survival analysis for TAM sensitivity- or resistance-related miRNA expression levels using TCGA and two independent GEO data sets

Overall survival was defined as the time from date of diagnosis to the date of death by any cause, and disease-free survival was defined as the time from date of diagnosis to the date of diagnosis of a metastatic breast cancer. OS and DFS were compared using the Cox proportional hazard model between the high and low expression groups determined by each miRNA-specific thresholds as described below. Stratified analyses were also conducted and the covariates in the models included the ER and TNBC status. In TCGA dataset, the clinical data and histological subtypes were determined using TNM staging and pathological molecular subtyping [39, 40].

### Cutoff point selection of high and low expression groups

Patients were placed into the low-expression or high-expression groups based on their miRNA expression levels in both TCGA and GEO cohorts. To determine the threshold of dichotomization, a running Cox proportional hazard statistic was applied [41]. Differences in the OS between the two groups were assessed at multiple candidate cutoff points within the range of risk score, and the optimal cutoff point was chosen based on the statistical significance of the Cox proportional hazards model.

### GSEA of each miRNA expression and TAM sensitivity- or resistance-related gene sets

GSEA was conducted on each miRNA and RNA expression data from TCGA to see if there was any association with gene sets reported to correlate to drug sensitivity or resistance in breast cancer. This was done using software provided by the Broad Institute (http://software.broadinstitute.org/gsea/index.jsp) [42]. GSEA was conducted only of TCGA dataset since the samples size were too small and we did not have access to RNA expression data from the other GEO datasets.

### Statistical analysis

All statistical analyses were performed using R software (http:///www.r-project.org/) and Bioconductor (http://bioconductor.org/). Data of miRNA expression was normalized using DESeq2 package [43] and log-transformed. Patients were split into low-expression and high-expression groups based on miRNA expression levels. A running Cox proportional hazard statistic was applied to determine the threshold of dichotomization [41]. To compare the survival curves of individual groups, the Kaplan-Meier method with log-rank tests and Cox proportional hazard models were used when appropriate. To test the proportional hazard assumption in Cox models, the Schoenfeld residuals test was used. Multivariable Cox proportional hazards regression analyses were utilized to evaluate independent prognostic value each factor. The reported results included hazard ratios (HR) and 95% confidence intervals (CI). A two-sided test with p<0.05 was considered statistically significant for the present analyses.

## SUPPLEMENTARY MATERIALS FIGURE AND TABLES



## References

[1] (2016). SEER Stat Fact Sheets: Female Breast Cancer. In: Institute NC, ed

[2] BREASTCANCER.ORG

[3] Parise CA, Caggiano V (2014). Breast cancer survival defined by the ER/PR/HER2 subtypes and a surrogate classification according to tumor grade and immunohistochemical biomarkers. J Cancer Epidemiol.

[4] Dai X, Li T, Bai Z, Yang Y, Liu X, Zhan J, Shi B (2015). Breast cancer intrinsic subtype classification, clinical use and future trends. Am J Cancer Res.

[5] Early Breast Cancer Trialists’ Collaborative Group (EBCTCG) (2005). Effects of chemotherapy and hormonal therapy for early breast cancer on recurrence and 15-year survival: an overview of the randomised trials. Lancet.

[6] Clarke R, Tyson JJ, Dixon JM (2015). Endocrine resistance in breast cancer--an overview and update. Mol Cell Endocrinol.

[7] Ma L, Teruya-Feldstein J, Weinberg RA (2007). Tumour invasion and metastasis initiated by microRNA-10b in breast cancer. Nature.

[8] Huang TH, Wu F, Loeb GB, Hsu R, Heidersbach A, Brincat A, Horiuchi D, Lebbink RJ, Mo YY, Goga A, McManus MT (2009). Up-regulation of miR-21 by HER2/neu signaling promotes cell invasion. J Biol Chem.

[9] Iorio MV, Ferracin M, Liu CG, Veronese A, Spizzo R, Sabbioni S, Magri E, Pedriali M, Fabbri M, Campiglio M, Menard S, Palazzo JP, Rosenberg A (2005). MicroRNA gene expression deregulation in human breast cancer. Cancer Res.

[10] Zhang K, Zhang Y, Liu C, Xiong Y, Zhang J (2014). MicroRNAs in the diagnosis and prognosis of breast cancer and their therapeutic potential (review). Int J Oncol.

[11] van Schooneveld E, Wildiers H, Vergote I, Vermeulen PB, Dirix LY, Van Laere SJ (2015). Dysregulation of microRNAs in breast cancer and their potential role as prognostic and predictive biomarkers in patient management. Breast Cancer Res.

[12] Mulrane L, McGee SF, Gallagher WM, O’Connor DP (2013). miRNA dysregulation in breast cancer. Cancer Res.

[13] Egeland NG, Lunde S, Jonsdottir K, Lende TH, Cronin-Fenton D, Gilje B, Janssen EA, Soiland H (2015). The role of microRNAs as predictors of response to tamoxifen treatment in breast cancer patients. Int J Mol Sci.

[14] Hayes EL, Lewis-Wambi JS (2015). Mechanisms of endocrine resistance in breast cancer: an overview of the proposed roles of noncoding RNA. Breast Cancer Res.

[15] O’Bryan S, Dong S, Mathis JM, Alahari SK (2017). The roles of oncogenic miRNAs and their therapeutic importance in breast cancer. Eur J Cancer.

[16] Muluhngwi P, Klinge CM (2017). Identification of miRNAs as biomarkers for acquired endocrine resistance in breast cancer. Mol Cell Endocrinol.

[17] THE CANCER GENOME ATLAS. https://cancergenome.nih.gov/

[18] Chin L, Andersen JN, Futreal PA (2011). Cancer genomics: from discovery science to personalized medicine. Nat Med.

[19] Enerly E, Steinfeld I, Kleivi K, Leivonen SK, Aure MR, Russnes HG, Ronneberg JA, Johnsen H, Navon R, Rodland E, Makela R, Naume B, Perala M (2011). miRNA-mRNA integrated analysis reveals roles for miRNAs in primary breast tumors. PLoS One.

[20] Buffa FM, Camps C, Winchester L, Snell CE, Gee HE, Sheldon H, Taylor M, Harris AL, Ragoussis J (2011). microRNA-associated progression pathways and potential therapeutic targets identified by integrated mRNA and microRNA expression profiling in breast cancer. Cancer Res.

[21] Wang Z, Gerstein M, Snyder M (2009). RNA-Seq: a revolutionary tool for transcriptomics. Nat Rev Genet.

[22] Ozsolak F, Milos PM (2011). RNA sequencing: advances, challenges and opportunities. Nat Rev Genet.

[23] Metzker ML (2010). Sequencing technologies - the next generation. Nat Rev Genet.

[24] Research Portfolio Online Reportng Tools (RePORT) http://projectreporter.nih.gov

[25] Miller TE, Ghoshal K, Ramaswamy B, Roy S, Datta J, Shapiro CL, Jacob S, Majumder S (2008). MicroRNA-221/222 confers tamoxifen resistance in breast cancer by targeting p27Kip1. J Biol Chem.

[26] Rao X, Di Leva G, Li M, Fang F, Devlin C, Hartman-Frey C, Burow ME, Ivan M, Croce CM, Nephew KP (2011). MicroRNA-221/222 confers breast cancer fulvestrant resistance by regulating multiple signaling pathways. Oncogene.

[27] Gan R, Yang Y, Yang X, Zhao L, Lu J, Meng QH (2014). Downregulation of miR-221/222 enhances sensitivity of breast cancer cells to tamoxifen through upregulation of TIMP3. Cancer Gene Ther.

[28] Wei Y, Lai X, Yu S, Chen S, Ma Y, Zhang Y, Li H, Zhu X, Yao L, Zhang J (2014). Exosomal miR-221/222 enhances tamoxifen resistance in recipient ER-positive breast cancer cells. Breast Cancer Res Treat.

[29] Cittelly DM, Das PM, Spoelstra NS, Edgerton SM, Richer JK, Thor AD, Jones FE (2010). Downregulation of miR-342 is associated with tamoxifen resistant breast tumors. Mol Cancer.

[30] He YJ, Wu JZ, Ji MH, Ma T, Qiao EQ, Ma R, Tang JH (2013). miR-342 is associated with estrogen receptor-alpha expression and response to tamoxifen in breast cancer. Exp Ther Med.

[31] Bergamaschi A, Katzenellenbogen BS (2012). Tamoxifen downregulation of miR-451 increases 14-3-3zeta and promotes breast cancer cell survival and endocrine resistance. Oncogene.

[32] Lowery AJ, Miller N, Devaney A, McNeill RE, Davoren PA, Lemetre C, Benes V, Schmidt S, Blake J, Ball G, Kerin MJ (2009). MicroRNA signatures predict oestrogen receptor, progesterone receptor and HER2/neu receptor status in breast cancer. Breast Cancer Res.

[33] Van der Auwera I, Limame R, van Dam P, Vermeulen PB, Dirix LY, Van Laere SJ (2010). Integrated miRNA and mRNA expression profiling of the inflammatory breast cancer subtype. Br J Cancer.

[34] Endo Y, Toyama T, Takahashi S, Yoshimoto N, Iwasa M, Asano T, Fujii Y, Yamashita H (2013). miR-1290 and its potential targets are associated with characteristics of estrogen receptor alpha-positive breast cancer. Endocr Relat Cancer.

[35] Masri S, Phung S, Wang X, Wu X, Yuan YC, Wagman L, Chen S (2008). Genome-wide analysis of aromatase inhibitor-resistant, tamoxifen-resistant, and long-term estrogen-deprived cells reveals a role for estrogen receptor. Cancer Res.

[36] Frasor J, Stossi F, Danes JM, Komm B, Lyttle CR, Katzenellenbogen BS (2004). Selective estrogen receptor modulators: discrimination of agonistic versus antagonistic activities by gene expression profiling in breast cancer cells. Cancer Res.

[37] Manolio TA, Fowler DM, Starita LM, Haendel MA, MacArthur DG, Biesecker LG, Worthey E, Chisholm RL, Green ED, Jacob HJ, McLeod HL, Roden D, Rodriguez LL (2017). Bedside back to bench: building bridges between basic and clinical genomic research. cell.

[38] Ramanathan R, Olex AL, Dozmorov M, Bear HD, Fernandez LJ, Takabe K (2017). Angiopoietin pathway gene expression associated with poor breast cancer survival. Breast Cancer Res Treat.

[39] Goldhirsch A, Wood WC, Coates AS, Gelber RD, Thurlimann B, Senn HJ (2011). Strategies for subtypes--dealing with the diversity of breast cancer: highlights of the St. Gallen International Expert Consensus on the Primary Therapy of Early Breast Cancer 2011. Ann Oncol.

[40] Sobin LH, Gospodarowicz MK, Wittekind C

[41] Crowley J, LeBlanc M, Jacobson J, Salmon S (1997). Proceedings of the First Seattle Symposium in Biostatistics Survival Analysis.

[42] Subramanian A, Tamayo P, Mootha VK, Mukherjee S, Ebert BL, Gillette MA, Paulovich A, Pomeroy SL, Golub TR, Lander ES, Mesirov JP (2005). Gene set enrichment analysis: a knowledge-based approach for interpreting genome-wide expression profiles. Proc Natl Acad Sci U S A.

[43] Love MI, Huber W, Anders S (2014). Moderated estimation of fold change and dispersion for RNA-seq data with DESeq2. Genome Biol.

