# Supplementary material for: Tamoxifen sensitivity-related microRNA-342 is a useful biomarker for breast cancer survival
Source: Oncotarget. 2017 Oct 6;8(59):99978–89. doi: 10.18632/oncotarget.21577 (PMC5725145; doi:10.18632/oncotarget.21577)
Supplement: Supplementary file 1 [file oncotarget-08-99978-s001.pdf]

Tamoxifen sensitivity-related microRNA-342 is a useful biomarker for breast cancer survival

SUPPLEMENTARY MATERIALS

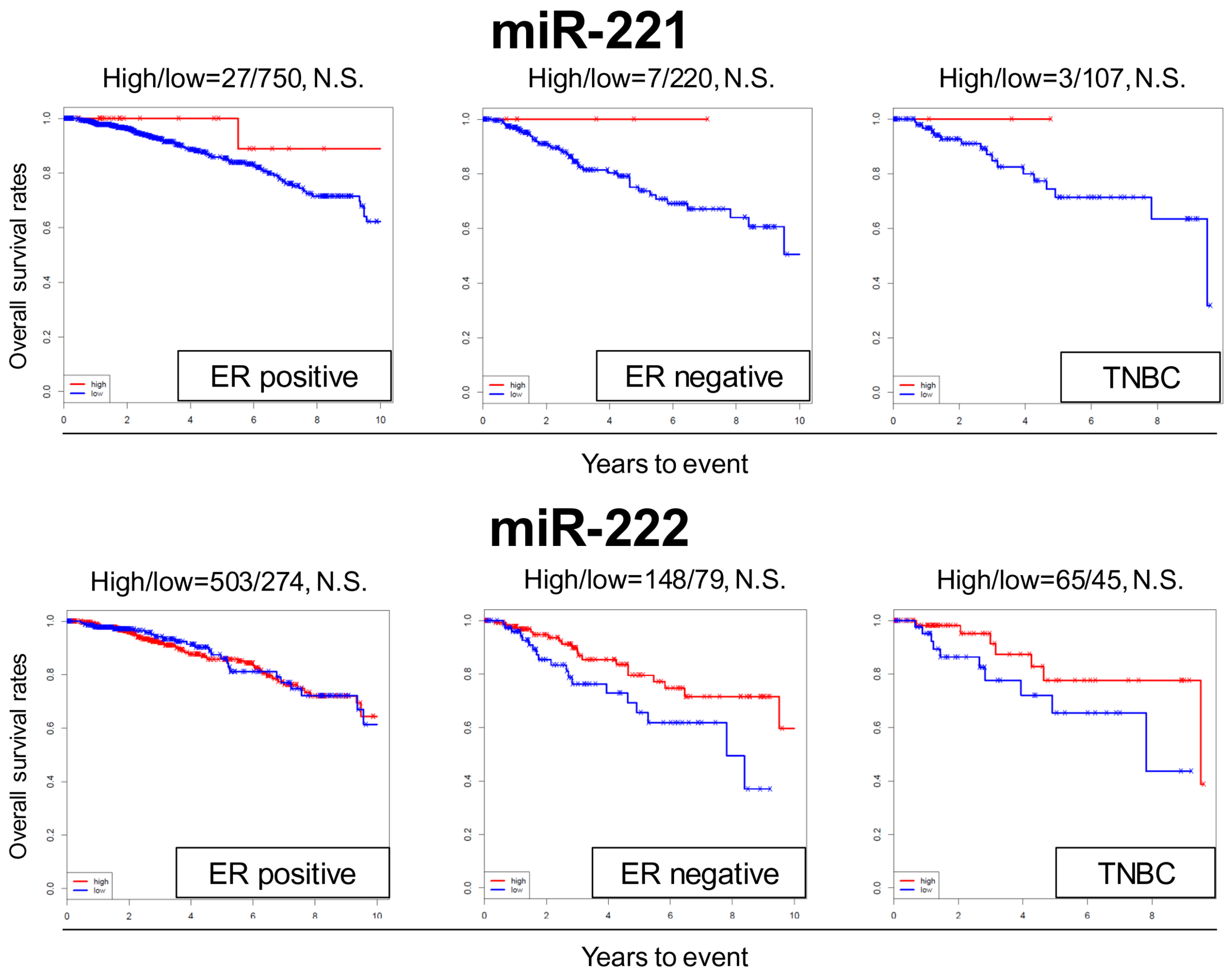

Supplementary Figure 1: Expression levels of miR-221 and miR-222 and survival by estrogen receptor status; ER-positive; ER-negative; and TNBC breast cancer patients in TCGA. High and low expressions are represented by the red and blue lines, respectively. Not significant (N.S.).

Supplementary Table 1: Association between clinical or pathological factors and miR-451 expression levels

	miR-451 [1]		P-value [2]
	High (n)	Low (n)	
Age <60	42	514	0.8704
>60	35	458	
Stage I/II/III/IV	14/46/16/1	164/562/227/19	0.9227
ER +/-	60/13	717/214	0.3800
PR +/-	51/22	624/306	0.7200
HER2 +/-	26/39	319/501	0.9700
TNBC Y/N	7/58	103/716	0.8184

[1] Cutoff point of this analyses is corresponding to optimal point derived from survival analyses.
[2] Chi square test.

Supplementary Table 2: Association between clinical or pathological factors and miR-221 expression levels

	miR-221 [1]		P-value [2]
	High (n)	Low (n)	
Age <60	17	539	0.3819
>60	21	472	
Stage I/II/III/IV	5/24/9/0	173/584/234/20	0.7345
ER +/-	27/7	750/220	0.9400
PR +/-	25/9	650/319	0.5500
HER2 +/-	7/20	338/520	0.2300
TNBC Y/N	3/24	107/750	1.0000

[1] Cutoff point of this analyses is corresponding to optimal point derived from survival analyses.
[2] Chi square test.

Supplementary Table 3: Association between clinical or pathological factors and miR-222 expression levels

	miR-222 [1]		P-value [2]
	High (n)	Low (n)	
Age <60	368	188	0.2344
>60	308	185	
Stage I/II/III/IV	112/390/162/12	66/218/81/8	0.8254
ER +/-	503/148	274/79	0.9600
PR +/-	439/212	236/116	0.9600
HER2 +/-	228/343	117/197	0.4800
TNBC Y/N	65/506	45/268	0.2368

[1] Cutoff point of this analyses is corresponding to optimal point derived from survival analyses.
[2] Chi square test.

Supplementary Table 4: Multivariable Cox proportional hazards regression analyses in TCGA cohort

Variable	HR[1]	95% CI[2]Lower	95% CI[2]Upper	P-value
miR-342	0.96	0.81	1.15	0.66
Age	2.54	1.65	3.91	<0.0001
Stage	2.60	1.94	3.48	<0.0001
ER	0.71	0.30	1.68	0.44
PR	0.73	0.38	1.41	0.35
HER2	0.91	0.55	1.50	0.70

[1] HR, hazard ratio; [2] CI, Confidence interval.
